# Theta Coherence Asymmetry in the Dorsal Stream of Musicians Facilitates Word Learning

**DOI:** 10.1038/s41598-018-22942-1

**Published:** 2018-03-15

**Authors:** Stefan Elmer, Joëlle Albrecht, Seyed Abolfazl Valizadeh, Clément François, Antoni Rodríguez-Fornells

**Affiliations:** 1 Cognition and Brain Plasticity Group, Bellvitge Biomedical Research Institute, L’Hospitalet de Llobregat, 08097 Barcelona, Spain; 2 Auditory Research Group Zurich (ARGZ), Division Neuropsychology, Institute of Psychology, University of Zurich, Zurich, Switzerland; 3 Sensory-Motor System Lab, Institute of Robotics and Intelligence Systems, Swiss Federal Institute of Technology, Zurich, Switzerland; 4 Department of Cognition, Development and Educational Psychology, Campus Bellvitge, University of Barcelona, L’Hospitalet de Llobregat, 08097 Barcelona, Spain; 5 Institució Catalana de Recerca i Estudis Avançats, ICREA, 08010 Barcelona, Spain; 6 Institut de Recerca Pediàtrica Hospital Sant Joan de Déu, Barcelona, Spain

## Abstract

Word learning constitutes a human faculty which is dependent upon two anatomically distinct processing streams projecting from posterior superior temporal (pST) and inferior parietal (IP) brain regions toward the prefrontal cortex (dorsal stream) and the temporal pole (ventral stream). The ventral stream is involved in mapping sensory and phonological information onto lexical-semantic representations, whereas the dorsal stream contributes to sound-to-motor mapping, articulation, complex sequencing in the verbal domain, and to how verbal information is encoded, stored, and rehearsed from memory. In the present source-based EEG study, we evaluated functional connectivity between the IP lobe and Broca’s area while musicians and non-musicians learned pseudowords presented in the form of concatenated auditory streams. Behavioral results demonstrated that musicians outperformed non-musicians, as reflected by a higher sensitivity index (d’). This behavioral superiority was paralleled by increased left-hemispheric theta coherence in the dorsal stream, whereas non-musicians showed stronger functional connectivity in the right hemisphere. Since no between-group differences were observed in a passive listening control condition nor during rest, results point to a task-specific intertwining between musical expertise, functional connectivity, and word learning.

## Introduction

In the last two decades, professional musicians have repeatedly been shown to serve as a reliable and powerful model for studying functional and structural plasticity in brain regions supporting auditory perception^1–5^, motor control^6–8^, and recently also higher cognitive functions^9,10^. However, such brain changes should not be considered as spatially isolated phenomena but rather as being part of intimately connected and mutually interacting neural networks^11,12^. This network perspective is supported, for example, by previous diffusion tensor imaging (DTI) studies demonstrating white matter differences (i.e., fractional anisotropy, radial diffusivity, or volume) between musicians and non-musicians in a substantial number of fiber tracts, including the arcuate fasciculus (AF)^13,14^, different subdivisions of the corpus callosum^15–17^, the corticospinal tract^18,19^ as well as the extreme capsule^20^.

Currently, there is striking evidence showing that both plastic changes in the auditory-related cortex (ARC) as well as altered neural network characteristics^15,21^ lead to remarkable behavioral advantages of musicians in processing a variety of speech cues manipulated in terms of voice-onset time^22–24^, pitch^25–28^, duration^22,23^, timbre^4,29^, rhythm^30^, and prosody^26,31^. However, these behavioral advantages do not seem to be restricted to auditory tasks but can likewise be observed in several cognitive domains, including attention^32^, short-term memory^33^, working memory^34^, and inhibition^10^. Although the specific origin of these advantages is not yet fully understood, it is supposed that shared neural networks, perceptual functions, and cognitive operations between the domains of speech and music may be one of the key features underlying cognitive facilitation^27,35,36^.

Music training has not only been shown to facilitate basic processing of speech sounds but also speech segmentation in adults and children^37–39^, one of the first steps of language learning that requires the ability to extract words from continuous speech. Furthermore, recently, Dittinger and colleagues^40^ took advantage of the multifaceted influence of music training, and investigated the neural signatures underlying word learning mechanisms in musically trained and untrained subjects^40,41^ and children undergoing music training^42^ while the participants learned the meaning of new words through word-picture associations of increased mnemonic complexity. Results from these studies showed a behavioral advantage of musicians and musically trained children in word learning, however, only when participants had to access semantic memory in order to judge whether new pictures were related to previously learned words. Accordingly, this behavioral superiority was accompanied by a shift of the N400 component from anterior to posterior scalp sites, possibly indicating a training-related facilitation in incorporating the newly-learned word-meaning associations into established lexical-semantic representations^43,44^.

Learning words of a new language requires the interplay between auditory perception, memory functions, and articulation^40,45^. Contemporary biological and linguistic^46–50^ models of speech processing conjointly postulate important computational differences between the ventral and dorsal processing streams. The ventral stream is bilaterally organized, stretches from pST and IP brain regions toward the temporal pole, and mediates the integration of phonetic entities into lexical and semantic representations^51^. By contrast, a left-lateralized dorsal stream projecting from pST and IP areas toward the frontal lobe contributes to the translation of the speech signal into articulatory representations^49^. However, it is noteworthy to mention that the dorsal stream does not exclusively support sensory-motor functions but is likewise recruited across different modalities during higher-level memory tasks^52–54^. In fact, previous MEG source-imaging studies reported that during both verbal^55^ and non-verbal^34,52,56^ tasks, brain activity in pST and IP regions as well as in Broca’s area was modulated as a function of working memory load. This perspective is also in line with clinical observations showing that patients with lesions encompassing the left AF often demonstrate impaired working memory functions^57–60^ and that intraoperative stimulation in awake brain tumor patients in the vicinity of the AF disrupts non-word repetition^61^. Furthermore, the dorsal stream has previously been shown to contribute to complex sequencing in the verbal domain^62^.

Nowadays, there is functional^63–65^ and anatomical^66,67^ evidence showing that the dorsal and ventral streams differentially contribute to word learning depending, among other factors, on the demands placed on sound-to-meaning and sound-to-articulation mapping mechanisms. By using a multimodal imaging approach, Lopez-Barroso and colleagues^67^ reconstructed the posterior, the anterior, and the long segment of the AF^68,69^, and revealed a positive correlation between functional and structural connectivity among Wernicke’s area and Broca’s territory (i.e., long segment of the AF) and the ability of the participants to remember pseudowords presented in the form of auditory streams. These results suggest that the learning of pseudowords leads to an increased recruitment the left dorsal stream, and that sensory-to-motor coupling mechanisms may contribute to generate the motor codes of new phonological sequences for facilitating verbal memory functions^49,70^. Otherwise, Catani and co-workers^66^ focused on the relationship between the degree of asymmetry of the long segment of the AF and verbal memory performance in a group of participants who learned word lists by using semantic strategies. In contrast to Lopez-Barroso and colleagues^67^, results revealed that individuals characterized by a more symmetric distribution of this fiber bundle were better at remembering the previously learned lexical items compared to those with a strong left-hemispheric asymmetry^66^. Finally, previous fMRI studies investigating the neural underpinnings underlying word learning mechanisms by using picture-word associations^64,71^ or visually presented sentences^72^, generally revealed increased brain activity in distributed neocortical areas situated along the two processing streams and accommodating lexical-semantic processes^47,51^, including the IP lobe, Broca’s area, and the middle-posterior part of the middle temporal gyrus (MTG).

**Figure 1:**
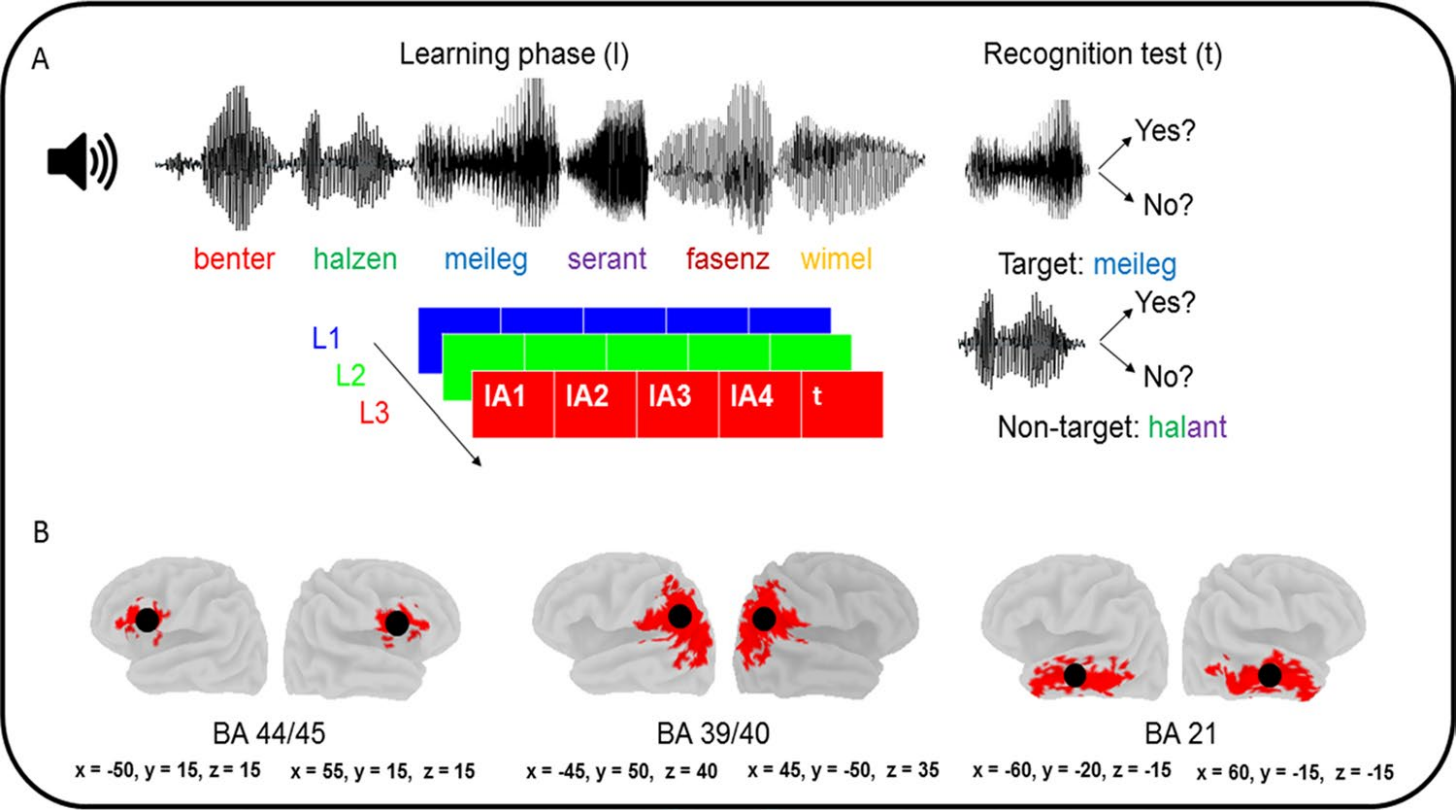
The upper part of the figure (**A**) provides an overview of the word learning paradigm consisting of different pseudoword lists (LA-LD) pseudorandomly presented in a serial order (L1-L3) during the “learning phase” (l). After the learning phase, participants performed the recognition test (i.e., t, “test phase”) consisting of judging whether the pseudowords have previously been presented in the “learning phase” (i.e., target) or not (i.e., non-target). The bottom part of the figure (**B**) indicates the spatial position of the ROIs in a canonical MNI template. The red spots indicate the voxels that constitute the BAs 44/45, 39/40, and 21, whereas the black circles show the approximate position of the centroid voxels used for connectivity analyses. x, y, and z = MNI coordinates of the centroid voxel.

In the present EEG study, we evaluated word learning mechanisms in musicians and non-musicians by relying on a similar paradigm that has previously been shown to recruit the left dorsal stream^67^. However, the novelty of our approach was that we evaluated dynamic electrophysiological coupling mechanisms between specific brain regions of the dorsal and ventral streams during word learning instead of focusing on white matter architecture, hemodynamic responses as an indirect marker of brain activity, or event-related potentials. Specifically, we collected scalp-EEG data while a group of musicians and non-musicians learned pseudowords auditorily presented in the form of concatenated speech streams (Fig. 1). Afterwards, the EEG signal was segmented in single epochs of 1 second, Fourier transformed, and subjected to functional connectivity (i.e., coherences) analyses in the source-space by using the eLORETA toolbox. Thereby, we used a hierarchical approach consisting of (1) collecting scalp EEG data, (2) validating the inverse-space solution, (3) selecting the frequency band and the regions of interest most reliably representing spectral-density distribution in the dorsal stream, (3) and assessing theta (θ) coherences (i.e., see the discussion section for a detailed description of θ) within the three-dimensional brain space. According to previous studies showing a positive relationship between functional connectivity in the left dorsal stream and word learning in musicians and non-musicians^41,67^ as well as on anatomical data indicating an optimization of the left dorsal stream as a function of music training^13,14^, we evaluated functional connectivity between the IP lobe and ventral part of the prefrontal cortex, and predicted that the behavioral advantage of musicians in word learning would be reflected by an increased left-hemispheric asymmetry.

## Results

### Autobiographical data, musical aptitudes, and cognitive capabilities

Separate t-test for independent samples did not reveal between-group differences in age, number of foreign languages spoken, or cumulative number of hours of foreign languages spoken. Furthermore, the two groups did not differ in terms of years of education or years of education of the parents (i.e., t-tests). As expected, the evaluation of musical aptitudes by means of a 2 × 2 ANOVA (i.e., 2 groups x 2 subtests) revealed main effects of group (F_(1, 28)_ = 63.054, p < 0.001) and subtest (F_(1, 28)_ = 23.89, p < 0.001) as well as a significant group x subtest interaction effect (F_(1, 28)_ = 5.465, p = 0.027). The main effect of group was related to an overall better performance of musicians compared to non-musicians (t_(28)_ = −8.731, p < 0.001), whereas the main effect of subtest as well as the group x subtest interaction were driven by a generally better performance in the rhythmical compared to the tonal condition (t_(28)_ = −4.55, p < 0.001), with a more pronounced discrepancy between the two subtests in non-musicians (i.e., t-test performed with difference values, t_(28)_ = 2.338, p = 0.027). Finally, none of the t-tests for independent samples targeting at screening for group differences in cognitive abilities (i.e., WIE, “cognitive speed”, and VLMT) reached significance.

### Behavioral data

**Figure 2:**
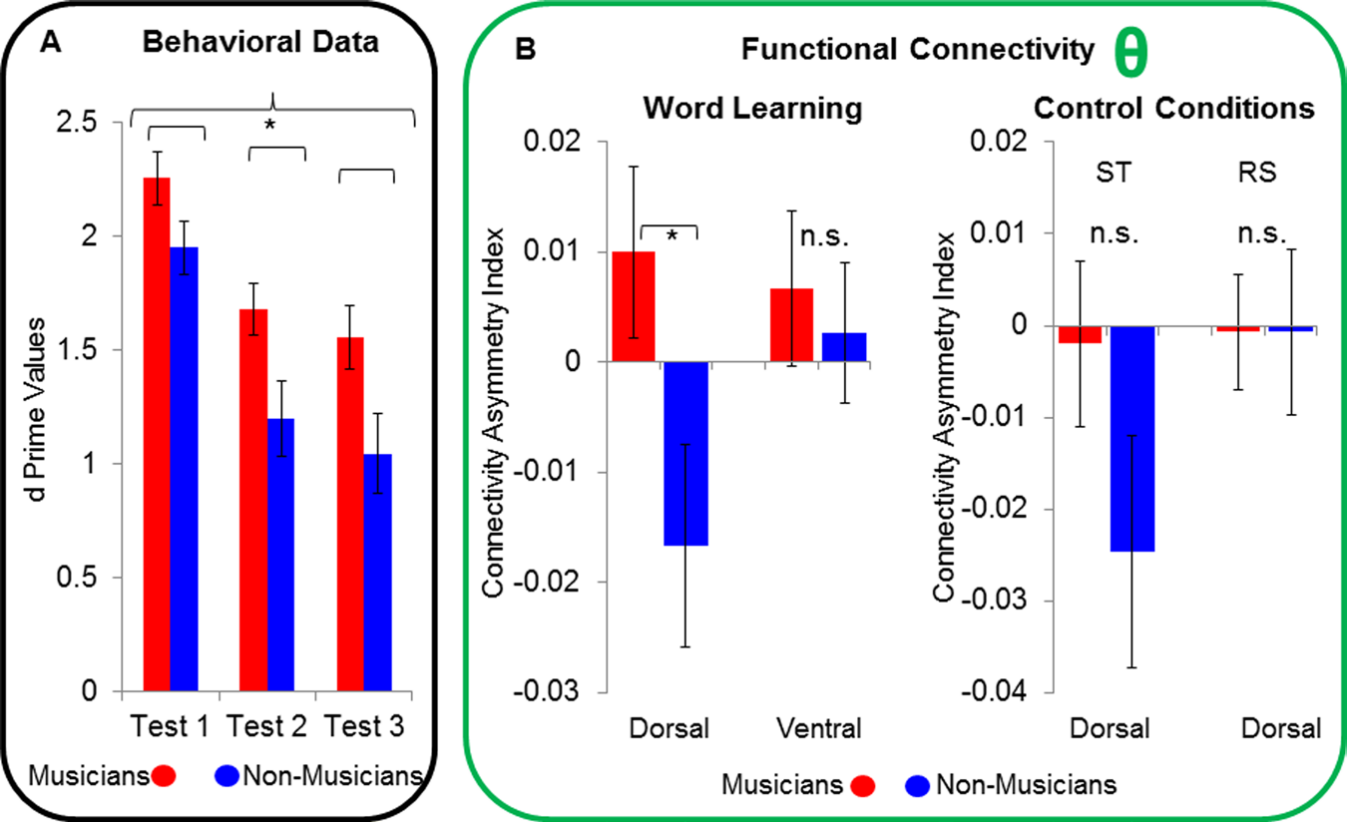
(**A**) shows d’ values, separately for the two groups (blue = non-musicians, red = musicians) and the three test blocks. The left part of (**B**) depicts between-group comparisons of the AI related to the dorsal and ventral streams during word learning. The right part of (**B**) shows between-group comparisons (i.e., red = musicians, blue = non-musicians) of the AI in the dorsal stream during the two control conditions, namely the Simon task (ST) and resting-state (RS). * = p < 0.05. n.s. = non-significant. The bars indicate standard error of mean.

The 2 × 3 repeated-measures ANOVA (i.e., two groups and three “test blocks”) computed on d’ scores yielded significant main effects of “test block” (F_(2, 27)_ = 49.767, p < 0.001) and group (F_(1, 28)_ = 6.433, p = 0.017). The main effect of “test block” originated from significantly higher d prime values in block 1 compared to block 2 and 3 (block 1 vs. block 2: t_(29)_ = 9.962, p < 0.001; block 1 vs. block 3: t_(29)_ = 7.664, p < 0.001), whereas the main effect of group was mediated by an overall better performance of musicians compared to non-musicians (Fig. 2A). All other effects did not reach significance (all ps > 0.05).

### Current-density reconstruction: localizer

**Figure 3:**
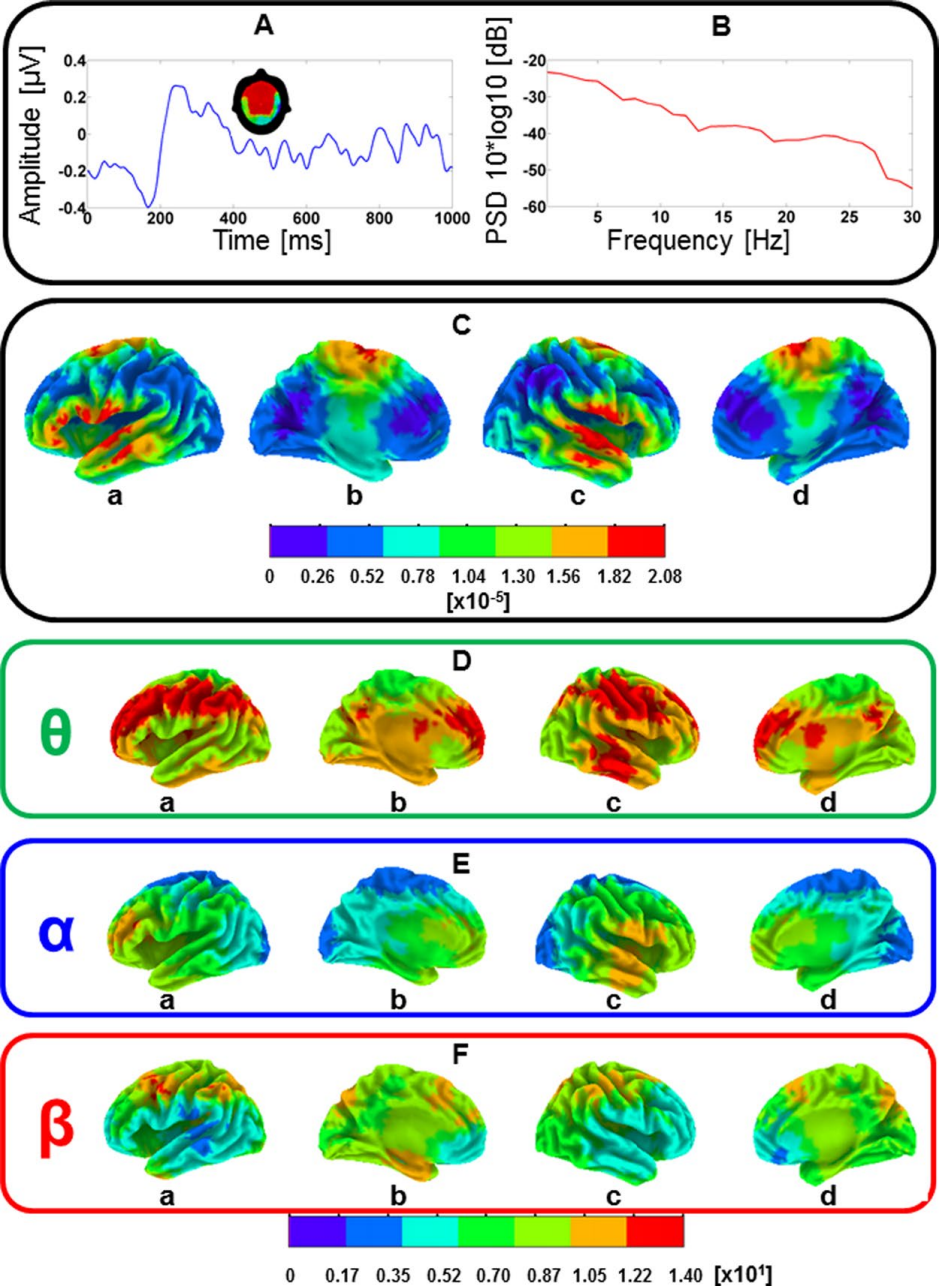
(**A**) average EEG activity across all electrodes, participants, and serial language positions. The scalp map indicates negative voltage values ranging from high (red color) to low (green color) negativity. (**B**) Power spectral density (PSD) across all electrodes, participants, and serial language positions. (**C**) Mean current-density distribution maps of the 1 second EEG segments averaged across participants and serial language positions in the range of 0–2 prop. µA/mm^2^ × 10^−5^. a = left lateral hemisphere, b = left medial hemisphere, c = right lateral hemisphere, d = right medial hemisphere. (**D**,**E**,**F**) SnPM results reflecting θ (green box), α (blue box), and low-β (red box) spectral-density distributions for all pseudoword lists (i.e., 1 second segments) averaged across the participants. The bottom bar (i.e., scaled in t units x 10^1^) depicts t-values (threshold for significance at p < 0.01, t = 3.35; one-tailed, corrected for multiple comparisons). a = left lateral hemisphere, b = left medial hemisphere, c = right lateral hemisphere, d = right medial hemisphere.

For validating the three-dimensional eLORETA source reconstruction, the single segments of 1 second were averaged across the three serial language positions and the two groups, and current density values were estimated for each voxel. This procedure clearly demonstrated an intuitive inverse-space solution, with maximal activity originating from the ARC (i.e., BA 42 and 22, see Fig. 3C).

### Spectral-density analyses

Statistical non-parametric mapping (SnPM) analyses computed in the θ, α, and low-β frequency-ranges brought to light three bilaterally distributed but spatially segregated spectral density maps. In particular, θ spectral-density was highest in frontal-parietal brain regions (i.e., including the IP lobe as well as the inferior frontal gyrus) and in the temporal lobe, whereas α was most pronounced at anterior brain sites with maximal spectral-density distribution in the proximity of the temporal pole and the middle and inferior frontal gyri. Otherwise, low-β spectral-density was highest in IP parietal- and posterior frontal regions, however, with lowest t-values around the sylvian fissure. Consequently, based on the fact that θ most reliably reflected spectral-density in the dorsal stream, connectivity analyses were computed in the θ frequency-band between the IP lobe and Broca’s area (see methods section and Fig. 1B). This approach is consistent with a previous MEG study focusing on functional connectivity in the dorsal stream during an auditory working memory task^52^.

### Functional connectivity analyses

Putative group differences in the degree of asymmetry of functional connectivity in the dorsal and ventral streams during the “learning phase” were evaluated by comparing the asymmetry indices (AI) between the two groups (Mann-Whitney U tests). Otherwise, for the two control conditions we only evaluated the AI in the dorsal stream (Mann-Whitney U tests).

### “Learning phase”

The Mann-Whitney U tests performed on the AI data revealed a significant group difference in the dorsal (U = 162, p = 0.041) but not in the ventral stream (U = 123, p = 0.683). As visible from Fig. 2B, musicians were characterized by a left-hemispheric asymmetry, whereas in non-musicians functional connectivity was higher in the right hemisphere.

### Control conditions

In order to exclude that the group difference we observed in the dorsal stream during the “learning phase” was possibly confounded by resting-state activity or induced by acoustic stimulation *per se* (i.e., Simon task), we performed two additional Mann-Whitney U tests with the AI data related to the dorsal stream. However, these analyses did not reach significance (Simon task: U = 149, p = 0.137; resting state: U = 101, p = 0.653; Fig. 2B, right side).

### Brain-training and brain-behavior relationships

Relationships between brain data, music training, and behavior were inspected by means of within-group correlative analyses (according to Spearman’s rho, one-tailed). In the musicians group we did not reveal a significant relationship between the AI in the dorsal stream during the “learning phase” and age of training commencement (p = 0.423). The same was the case for the correlation between the former variable and the cumulative number of training hours across lifespan (p = 0.093). Furthermore, within both groups, the AI of the “learning phase” did not correlate with d’ (musicians: p = 0.39; non-musicians: p = 0.113).

## Discussion

### Behavioral data

In the present study, we specifically evaluated a homogeneous group of musicians consisting of pianists. In fact, this specific genre of musicians has previously been shown to be more prone to plastic changes in the left hemisphere, whereas musicians playing instruments producing less sharp and impulsive tones (e.g., bassoon, saxophone, French horn, violoncello, or organ) are more likely characterized by neural adaptations in the right counterpart^5^. According to the signal detection theory^73^ which allows to measure individual discrimination sensitivity (d’), musicians showed higher d’ values compared to non-musicians. This result clearly demonstrated a superiority of musicians in learning new words from concatenated speech streams. The behavioral results also uncovered a main effect of test block that originated from a decay of performance from test block 1 to block 2 and 3. Since this effect was not influenced by musical expertise, the data are interpreted as indicating a general interference effect originating from repeated filler stimuli (i.e., non-target stimuli).

Our behavioral results are compatible with a previous EEG study that investigated speech segmentation abilities in children undergoing music training^37^. Thereby, François and colleagues^37^ revealed that during the “test phase” musically trained children showed higher familiarity accuracy than children undergoing painting training for pseudowords that have previously been presented in the form of concatenated streams with high transitional probabilities (i.e., pseudowords vs. partial pseudowords). However, improved speech segmentation skills as a function of music training might not be the sole effect contributing to the present results. In fact, Dittinger and colleagues evaluated word learning mechanisms through picture-word associations in children undergoing music training^42^ and professional musicians^40,41^ compared to untrained participants, and revealed that musically trained individuals were better able to learn the meaning of new words differing in pitch, duration, voice-onset time, and aspiration. Accordingly, the authors proposed that at least two mechanisms can lead to a behavioral advantage of musicians in word learning, namely enhanced phonetic perception^23,31^ and a general improvement of cognitive functioning^10,74^. Here, musicians and non-musicians did not differ in terms of general cognitive functioning (i.e., IQ, short-term memory, and working memory). Consequently, the behavioral advantage we revealed in musicians during word learning might rather be explained by superior phonetic perception abilities and segmentation skills than in terms of a general optimization of cognitive functions. In particular, as previously suggested by Dittinger and colleagues^40^, it is conceivable that enhanced phonetic perception in musicians may facilitate the building of new phonological representations (i.e., through the recruitment of the left dorsal stream) that are necessary for word segmentation, leading to more stable mnemonic representations.

### Frequency-band selection and ROIs

Statistical spectral-density analyses showed that θ oscillations best reflected consistent spatial distribution patterns in the dorsal stream. In fact, α and low-β generators were spatially dissociated from θ current-densities, and were most prominently distributed in anterior-temporal- and frontal areas (α) as well as in the IP lobe and superior frontal brain regions (low-β). Furthermore, low-β activity was generally decreased in perisylvian territories and in the ventral frontal cortex. Interestingly, the spatial distribution of θ spectral-density (see Fig. 3D) showed a strong similarity with the dorsal network recently extracted by Albouy and colleagues by means of MEG during a pitch memory task^52^ and corroborates also previous findings relating theta oscillatory activity with word learning^75^. However, this spatial convergence may not be surprising since theta oscillations have repeatedly been linked to a variety of verbal^76,77^ and non-verbal^78^ memory functions, including short-term memory^79^, working memory^52^ as well as episodic memory^80^. From a physical perspective, low-frequency oscillations are characterized by high amplitudes and long wavelengths making them particularly suitable for integrating information between spatially dislocated brain areas^81^ as well as for coordinating neuronal communication over long-range circuits^81,82^. Furthermore, theta oscillations have previously been shown to play to an important role in “packing” the multi-time speech signal into units of the appropriate temporal granularity^83^ as well as to contribute to the temporal alignment of neural activity between the speech perception and production systems^84^. Increased coherence in the θ band (4–8 Hz) has also been observed in participants who learned new words in the context of a speech segmentation task^75^. This result also fits well with other studies showing a gradual increase in θ power and coherence during the progressive building up of working memory traces of linguistic information during sentence comprehension^85–87^. Finally, increased long-range fronto-parietal θ coherence has been reported in human studies involving periods of information retention and has been attributed to a common mechanism of neural interactions that sustains working memory functions^88^.

According to the θ spectral-density maxima distributed along the dorsal stream, the ROIs used for connectivity analyses were centered in the IP lobe and in Broca’s area (i.e., see methods section). The IP lobe has repeatedly been associated with higher-level phonetic processing, especially during tasks requiring the integration of acoustic information into short-term memory for later comparisons^89–91^. In addition, the IP lobe is known to usually be more strongly activated while processing pseudowords compared to real words^92^, and gray and white matter volume in this brain region has been shown to be predictive of speech sounds learning^93^. Although it is apparent that the spatial location of our posterior ROI diverged from the one used by Lopez-Barroso *et al*.^67^, the two approaches are thoroughly reconcilable from an anatomical perspective. In fact, it is well documented that the AG/SMG constitute a “plie de passage” of the long segment of the AF which runs through the temporal-parietal boundary for reaching Broca’s regions^68,69^. By contrast, the ROI centered in Broca’s area is fully comparable with this previous work^67^. Currently, it is generally recognized that domain-general and language-selective functions lay side-by-side within Broca’s area^94^, a territory that has repeatedly been associated with the planning and execution of speech articulation^95^, syntactic-, lexical-, semantic-, and phonological processes^47,96^, as well as with a variety of verbal working memory functions^94^. In addition, Broca’s area seems to be particularly responsive to pseudowords presented in isolation^92^ or in the context of concatenated speech^67^.

### Functional connectivity during the “learning phase”

In line with our hypothesis, connectivity analyses yielded a group difference in the dorsal but not in the ventral stream that originated from increased left-hemispheric asymmetry in musicians compared to non-musicians, whereas non-musicians showed a shift toward the right hemisphere. These results are not only consistent with previous functional^11^ and anatomical^13,14^ studies pointing to an optimization of this left-sided neural circuit in musicians, but also with recent data attributing a fundamental role to the AF in mediating audio-motor learning^97^. In this context, it results self-explanatory that such sensory-to-motor coupling mechanisms are a fortiori triggered in musicians^8,98^ due to the continuous adjustment of motor output as a function of auditory feedback. The neural basis of this phenomenon has previously been described in professional musicians by evaluating directional EEG-based intracranial functional connectivity between the ARC and the premotor cortex^99^. Thereby, unidirectional impulse propagation from the ARC toward the premotor cortex was considerably stronger during piano playing compared to rest. Accordingly, the increased left-hemispheric asymmetry we revealed in musicians suggests that the participants performed the word learning tasks by more strongly reverting to this highly-trained and automated neural mechanism, probably contributing to facilitate keeping phonetic information in verbal memory through sensory-to-motor coupling mechanisms. This perspective is also anchored on a recent study of Tian and colleagues^100^ showing that articulation-based- (ABS) and hearing-based memory (HBS) strategies dissociate within the dorsal stream. In particular, ABS induced increased activity in frontal-parietal sensorimotor systems, whereas HBS was dependent on brain regions known to be implicated in memory retrieval, including the middle frontal gyrus, the IP lobe as well as the intraparietal sulcus^100,101^. Consequently, our results are interpreted as pointing to an optimized neural synchronization between brain regions involved in merging syllabic- and articulatory representations, and contributing to facilitate the building-up of more robust multidimensional memory traces^102^.

Interestingly, in non-musicians we did not reveal a comparable left-hemispheric neural preference. Although this might be seen somewhat in contrast to the previous work of Lopez-Barroso and colleagues^67^, it is noteworthy to mention that the authors principally found a correlation between left-hemispheric functional and structural connectivity and behavioral performance. Therefore, by taking into account such a linear relationship, our results are thoroughly comparable with these previous data in that the participants who were characterized by a stronger left-hemispheric asymmetry (i.e., the musicians) performed better on the word form learning tasks. However, we are more likely prone to interpret the observed between-group differences by taking into account differential learning strategies^103^. Based on the fact that sensory-to-motor coupling mechanisms have exclusively been attributed to the left hemisphere, the stronger right-sided engagement of the dorsal stream in non-musicians may rather reflect stronger demands placed on working-^104^ and episodic^105,106^ memory functions. This argumentation can be deduced from previous meta-analyses conducted with large samples and pointing to the involvement of widespread bilateral frontal-parietal networks during a variety of working memory task^107,108^ as well as while encoding auditory events in episodic memory^105,106^. In addition, working- and episodic memory have been shown to spatially overlap in frontal and parietal brain regions^109,110^, leading to suggest a mutual interdependence between these two memory systems during word learning^45^. Alternatively, we cannot neglect that the stronger right-sided engagement of the dorsal stream we revealed in non-musicians may possibly also have been induced by a specific task strategy consisting of anchoring pseudowords onto already established lexical-semantic representations. This view is, at least in parts, supported by a previous anatomical study showing a relationship between the degree of symmetry of the AF and lexical-semantic memory functions^66^ as well as by numerous studies clearly demonstrating that the IP lobe and Broca’s area are involved in mediating lexical-semantic access at both the word- and sentence level^47,48,111^.

### Brain-behavior relationships

In contrast to Lopez-Barroso and co-workers^67^, we did not reveal significant correlations between the degree of functional asymmetry in the dorsal stream (i.e., AI during the “learning phase”) and behavior (i.e., d’). Therefore, we presume that possibly a third latent variable that has not been evaluated in the present study may have mediated the brain effects to behavior. Such a latent variable could, for example, be detected by using more sophisticated data-driven procedures, like small-world network analyses or machine learning algorithms, instead of focusing on a-priori defined hypotheses. The second unexpected finding was that within the musicians group we did not reveal significant relationships between brain data (i.e., AI) and training parameters (i.e., age of commencement and cumulative number of training hours), letting open the possibility that the differential recruitment of the dorsal stream we observed between the two groups possibly reflected the influence of domain-specific predispositions rather than experience-dependent brain changes. Such a perspective is thoroughly conceivable in that previous MRI studies have shown that genetic factors have a significant influence on grey matter parameters in Broca’s area and in the IP lobe^112^, and that functional connectivity between these two brain regions can be inherited^113^. Finally, one other possible reason for not having found a correlation between the AI and training parameters is that the task we used was not sensitive enough to capture such a relationship. Certainly, for definitively answering this question future studies should make use of longitudinal designs enabling to capture the causal relationship between music training and functional connectivity.

## Conclusions

By using a previously tested experimental procedure^67,114^, we were able to uncover a task-specific relationship between the stronger left-asymmetric recruitment of the dorsal stream in the θ frequency range in musicians and word learning. Results are interpreted as suggesting that an optimization of sensory-to-motor coupling mechanisms and phonological working memory functions enabled the building-up of more robust multimodal memory traces. However, since we did not reveal significant correlations between brain data and training parameters, results may rather indicate the influence of predisposition on brain maturation rather than experience-determined brain changes. Our results may be considered as a first step toward a better understanding of the functional role of θ oscillations in the left dorsal stream as well as of its association with word learning, and open novel insights into the neural dynamics underlying foreign (but phonologically similar) word learning. Nevertheless, it is important to mention that EEG-based functional connectivity only enables an approximation of the neural sources contributing to word learning with a low spatial resolution. Therefore, the ROIs used for connectivity analyses should not be interpreted in an anatomical manner and the data should rather be interpreted as reflecting a coupling between anterior and posterior brain areas with a certain degree of dispersion.

## Materials and Methods

### Participants

Fifteen professional musicians and 15 non-musicians in the age range of 20–40 years and with no past or current neurological, psychiatric, or neuropsychological disorders participated in the study. All participants were consistently right-handed^115^ native German speakers, and none of them was bilingual. The musicians group consisted of 15 pianists (7 female; mean age = 26.53 years, SD = 5.23; mean age of training commencement = 7.27, SD = 2.25, mean cumulative number of training hours across lifespan = 18799.47, SD = 10776.5) with a conservatory degree or who were advanced students at the same institution. By contrast, the control group was composed of 15 volunteers without formal musical education (8 female; mean age 24.6 years, SD = 4.85). Furthermore, the non-musicians did not learn to play a musical instrument and did not play or sing in bands. In order to exclude between-group differences in terms of demographic variables and social economic status musicians and non-musicians were also matched in terms of years of education as well as years of education of the parents. The participants were paid for participation, the local ethics committee (i.e., Kantonale Ethikkommission Zurich) approved the study (in accordance with the Helsinki declaration), and written informed consent was obtained from all participants.

### Pure tone audiometry

All participants were tested with pure-tone audiometry (MAICO Diagnostic GmBh, Berlin) in the frequency-range of 250–8000 Hz (MAICO Diagnostic GmBh, Berlin). According to this procedure, all participants demonstrated an unremarkable audiological status (i.e., all tested frequencies could be heard below a threshold of 30 dB).

### Musical aptitudes, history of music training, and foreign language competence

Musical aptitudes were estimated by means of the “Advanced Measure of Music Audition” (AMMA) test^116^. This test consisted of 30 successive trials in which subjects had to compare pairs of piano melodies, and to decide whether the melodies were equivalent, rhythmically different, or tonally different. Autobiographical data on the history of music training as well as on the usage of foreign languages were collected by using an in-house questionnaire^2^.

### Cognitive capabilities

A set of standardized psychometric tests was used to compare a wide spectrum of basic cognitive functions between the two groups. Intelligence was assessed by means of four subtests^117^ of the WIE test battery^118^, including (1) “number-symbol associations”, (2) “commonalities finding”, (3) “mosaic test”, and (4) “digit span forward and backward”. Shortly, the “number-symbol association” test enables to estimate non-verbal associative memory functions, and consists of associating geometric patterns with numbers as fast as possible. During the “commonalities finding” procedure targeting at testing semantic memory functions, participants had to mention the generic term that coalesced the meaning of two words (e.g., musical instrument for the words piano and drum). The “mosaic test” was used to assess visual-spatial abilities and consisted of assembling cubes according to a predetermined visual template. Finally, during the “digit span forward” (i.e., short-term memory) and “backward” (i.e., working memory) tests the participants had to overtly reproduce sequences of digits of increased length. The raw values of these four subtests were transformed to a standardized composite T-value previously shown to adequately capture general intellectual abilities^117^. In addition, all participants performed a verbal memory (VLMT) test^119^ consisting of remembering as many auditory-presented words as possible from a pool of fifteen items as well as a “cognitive speed” test^120^ requiring to link serial numbers as fast as possible.

### Auditory stimuli

The pseudowords used in the present study consisted of disyllabic and low-associative items that were assembled according to German phonotactic constraints^121^. These auditory stimuli (i.e., totally 80, 40 presented in the “learning phase” and 40 presented as distractors during the “test phase”) were recorded by a native female speaker with a sample rate of 44.1 KHz and by using a two-channel record device (Teac Professional, DR-05). For each item, fundamental frequency (*f*0) was assessed by using the Praat software (http://www.fon.hum.uva.nl/praat/), and set to a timely-constant value of 200 Hz in order to eliminate pitch fluctuations over time (i.e., flattened speech). Furthermore, 20 ms logarithmic fade-in and fade-out were applied for avoiding an abrupt onset and decay, and amplitudes were normalized to a mean value of −23 dB by using the Adobe Audition software (Version 3.0, http://www.adobe.com/ch_de/products/audition.html). Afterwards, for each of the four pseudoword lists, 10 disyllabic pseudowords were concatenated as auditory streams (i.e., four different pseudo-randomized versions were presented in the four blocks of the “learning phase”) with a brief pauses of 25 ms inserted between the single words (Praat, http://www.fon.hum.uva.nl/praat/) for marking words boundaries^67,114^. This procedure resulted in three naturally spoken pseudoword lists (i.e., single word duration ~ 500–600 ms).

### Experimental procedure

During the “learning phases” (i.e., each of them consisting of four learning blocks, total duration of about 3 minutes), the pseudoword lists were successively presented in the form of auditory streams, and participants had to memorize as many pseudowords as possible (see Fig. 1A). During each of the four learning blocks, the same 10 disyllabic items were presented 5 times, however, in a pseudo-randomized order (i.e., same order for all participants, the same word was never repeated in succession). Furthermore, in order to minimize interferences between the four pseudoword lists, each syllable was uniquely presented within but not across languages. For example, if the word “benter” was part of the first language, than neither the syllables /ben/ nor /ter/ was part of the other languages. After the “learning phase”, participants started the “test phase” (i.e., duration of about 1 minute), consisting of judging (i.e., via a button press) whether each single auditorily presented item has previously been presented in the “learning phase” or not. For each pseudoword list, the “test phase” consisted of 3 blocks in which all learned items as well as non-learned items (i.e., 50%, distractors) were presented 3 times (i.e., once in each block). The distractors consisted of the same syllables as the learned items but were arranged in a scrambled manner. For example, if the words “benter” and “halzen” were learned items, then the word “zenben” was a possible distractor. Two minutes after the “test phase”, the next pseudoword list (i.e., “learning phase”) was presented. The serial order (i.e., L1-L3) of the four pseudoword lists (i.e., LA-LD) was pseudo-randomized within the two groups according to the following sequences: (1) LA-LB-LC, (2) LB-LA-LD, (3) LC-LD-LA, (4) LD-LC-LB. The auditory stimuli were delivered by using in-ear headphones (Sennheiser, CX 350) at an intensity of 70 dB, and stimulus presentation was controlled by the “Presentation” software (Neurobehavioral Systems, https://www.neurobs.com).

### Experimental control conditions

At the beginning and at the end of the word learning paradigm, the participants were additionally tested on two control conditions used for corroborating the specificity of the EEG effects observed during word learning. The first control condition consisted of a resting-state measurement of 3 minutes (i.e., eyes closed), and targeted at excluding between-group differences in intrinsic functional connectivity^11^ that might have interacted with brain oscillations during word form learning. By contrast, the second control condition was used for excluding that between-group differences during pseudoword learning were spuriously driven by acoustic stimulation per se. With this purpose in mind, at the end of the experiment, all participants were additionally exposed to the four pseudoword lists (i.e., pseudo-randomized within the two groups) while performing a Simon task^122^. This specific task relies on conflict monitoring functions^122^, and is known to dissociate from activity in the dorsal stream by principally recruiting anterior cingulate areas^123^. During the Simon task, participants sat in front of a computer monitor, and blue and red boxes were presented on the left or right side of the screen. Participants were instructed to press the button on the right when they see the red box appear on the screen and the button on the left when they see the blue one while at the same time ignoring acoustic stimulation. In such an experimental condition, reaction times are usually faster when the spatial position of the boxes (i.e., left or right) is congruent with the location of the response button (i.e., congruent spatial stimulus-response compatibility). Due to a technical problem with the response box, the behavioral data of one subject of the control group could not be collected.

### EEG data acquisition and pre-processing

Continuous EEG (32 electrodes, provided by Easy Cap) was recorded with a sampling rate of 1000 Hz and a high-pass filter of 0.1 Hz by using an EEG amplifier (Brain Products). The electrodes (sintered silver/silver chloride) were located at frontal, temporal, parietal, and occipital scalp sites according to the international 10–10 system (Fp1, Fp2, F7, F3, Fz, F4, F8, FT7, FC3, FCz, FC4, FT8, T7, C3, Cz, C4, T8, TP9, TP7, CP3, CPz, CP4, TP8, TP10, P7, P3, Pz, P4, P8, O1, Oz, and O2). The reference electrode was placed on the tip of the nose, and impedances were reduced to 10 kΩ by using electro-gel. For all pre-processing steps, we used the Brain Vision Analyzer software package (version 2.01; Brain Products). Data were filtered off-line with a low-pass filter of 30 Hz (i.e., including a Notch filter of 50 Hz), and artifacts (i.e., eye movements and blinks) were corrected by using an independent component analysis^124^ in association with a semi-automatic raw data inspection. After data pre-processing, the “resting-state” period, the “learning phase”, as well as the “Simon task” were segmented into single sweeps of 1000 ms, and subjected to eLORETA toolbox for analyses.

### Current-density source estimation: localizer

In a first hierarchical processing step, we validated the eLORETA (eLORETA software package; http://www.uzh.ch/keyinst/loreta.htm) source estimation approach by demonstrating current-density maxima originating from the ARC across pseudoword lists. The single artifact-free EEG segments of all the “learning phases” (i.e., segments of 1 second) were averaged for each participant and across the two groups and subjected to eLORETA source estimation. The eLORETA approach, unlike conventional dipole fitting, does not require a-priori assumptions about the number and the localization of the dipoles. eLORETA calculates the three-dimensional distribution of electrically active neuronal generators in the brain as a current density value (µA/mm^2^), and provides a solution for the inverse problem by assuming that the smoothest of all possible activity distributions is the most plausible one for explaining the data. The characteristic feature of this particular inverse solution approach is the low spatial resolution, which conserves the location of maximal activity, but with a certain degree of dispersion^125^.

In the current implementation of eLORETA, computations were made within a realistic head model^126^ by using the Montreal Neurological Institute (MNI) 152 template^127^, with a three-dimensional solution restricted to cortical gray matter, as determined by the probabilistic Talairach atlas^128^. The intracranial volume is partitioned in 6239 voxels at 5 mm spatial resolution. eLORETA images represent the electric activity at each voxel in the neuroanatomic MNI space as the magnitude of the estimated current density. Anatomical labels and Brodmann areas (BA) are reported using MNI space, with correction to Talairach space^129^.

### Spectral-density statistics

In a second hierarchical step, we evaluated eLORETA-based spectral-density maps in the theta (θ, 4–7 Hz), alpha (α, 8–12 Hz), and low-beta (β, 13–20 Hz) frequency-range, across all pseudoword lists, by means of voxel-by-voxel t-tests for zero-mean^130^. The decision to focus on the lower-β band was motivated by previous EEG studies showing an association between this frequency-range and word consolidation^131^ and word learning^41^. We used a statistical non-parametric mapping (SnPM) procedure that (1) has previously been shown to generate spatial results overlapping with fMRI results^130^, (2) enables to estimate the probability distribution by means of randomization statistics (i.e., 5000 permutations), and (3) is corrected for multiple comparisons with a high statistical power^132^. This procedure was used for selecting the frequency band of interest best reflecting activity in the dorsal stream^52^. In particular, we used a threshold of p < 0.01 (one-tailed) and only tested for current-density values above average (i.e., one-tailed) because we reasoned that the specificity of brain regions responsive to word learning should be reflected in increased and not decreased EEG activity. For each participant, the single sweeps of 1000 ms related to the “learning phase” were Fourier transformed in the a-priori defined frequency-ranges and averaged. Afterwards, eLORETA images corresponding to the estimated neuronal generators of brain activity within a given frequency band (i.e., for each voxel)^133^ were computed and subjected to SnPM analyses (i.e., p < 0.01, corrected for multiple comparisons). For SnPM analyses, the estimated spectral-density values for each voxel in the whole sample of participants were subjected to voxel-by-voxel comparisons by using the eLORETA toolbox. By using a t-test for zero-mean, the spectral density values of each voxel were statistically compared to the average spectral density distribution. This procedure enables to calculate t-values for each voxel through permutation statistics according to a critical value of p < 0.01.

### Functional connectivity analyses

Functional connectivity (eLORETA software package; http://www.uzh.ch/keyinst/loreta.htm) was evaluated by using lagged coherence values as a measure of the variability of two signals in a specific frequency band^134^. This nonlinear functional connectivity measure was calculated based on normalized Fourier transforms for each voxel, and is corrected in order to represent the alignment between two signals after the instantaneous zero-lag contribution has been excluded^135,136^. Such a correction is warranted because zero-lag connectivity in a given frequency band is normally due to non-physiological effects or intrinsic physical artifacts.

In the current implementation of the eLORETA software, computations are made within a realistic head model^126^ relying on the Montreal Neurological Institute (MNI) 152 template^127^. Based on the results of the SnPM statistics (Fig. 3D–F), functional connectivity was computed in the θ frequency-range between frontal and parietal brain regions, and the ROIs were centered in BA 39/40 and BA 44/45. Since eLORETA has a low spatial resolution that does not enable to distinguish between anatomically adjacent brain regions, these ROIs were selected based on (1) a previous fMRI study that used a similar word learning paradigm and showed activity in posterior perisylvian and IP (i.e., roughly corresponding to BA 39/40) brain regions as well as in Broca’s area (i.e., roughly corresponding to BA 44/45)^67^, (2) previous anatomical studies describing a direct white matter projection between the IP lobe and the ventral part of the prefrontal cortex^68,69^, as well as (3) on a recent EEG study that evaluated word learning in musicians and non-musicians by using a similar inverse-space solution as the one used in the present work^41^. Furthermore, functional connectivity between the BA 39/40 and BA 21 was used as a control condition. The latter brain region was chosen according to current models of speech processing postulating its contribution to lexical-semantic access at the word level^47,49,111^. For functional connectivity analyses (see Fig. 1B), a method using a single voxel at the centroid of the ROIs was chosen^137^. Mathematical details on eLORETA functional connectivity algorithms can be found elsewhere^138^. Finally, it is important to remark that EEG-based functional connectivity data are always a composite of signal of interest and noise and this method does not preclude that functional connectivity between two regions of interest is indirectly mediated via a non-evaluated connection^139^.

### Statistical analyses

The analyses of autobiographical-, psychometric-, and behavioral data were performed by using t-tests for independent samples (i.e., two-tailed) and omnibus analyses of variance (i.e., ANOVA, repeated measurements). Since coherence measures in small samples deviate from normal distribution^140^, we tested putative group x hemisphere interactions in the dorsal and ventral streams by using non-parametric Mann-Whitney U tests. In particular, we computed separate Mann-Whitney U tests for the dorsal and ventral streams, and compared functional connectivity differences (i.e., left minus right = asymmetry index = AI) between the two groups. Thereby, we evaluated mean connectivity values across serial language positions. Furthermore, based on specific a-priori hypotheses, brain-behavior relationships were assessed by means of one-tailed correlative analyses according to Spearman’s rho. In particular, we expected to find a positive relationship between the amount of music practice and functional connectivity (i.e., AI) as well as between functional connectivity and behavioral data.
